# Revision of J3Gen Validity of the Attacks by Peinado *et al.*

**DOI:** 10.3390/s150511988

**Published:** 2015-05-22

**Authors:** Alberto Peinado, Jorge Munilla, Amparo Fúster-Sabater

**Affiliations:** 1 Escuela Técnica Superior de Ingeniería de Telecomunicación, Universidad de Málaga, Málaga 29071, Spain; E-Mail: apeinado@ic.uma.es; 2 Instituto de Tecnologías Físicas y de la Información, Consejo Superior de Investigaciones Científicas, Madrid 28006, Spain; E-Mail: amparo@iec.csic.es

**Keywords:** pseudo random number generators, security, cryptanalytic attack, Radio Frequency Identification, EPCglobal Gen2

## Abstract

This letter is the reply to: Remarks on Peinado *et al.*’s Analysis of J3Gen by J. Garcia-Alfaro, J. Herrera-Joancomartí and J. Melià-Seguí published in *Sensors* **2015**, *15*, 6217–6220. Peinado *et al.* cryptanalyzed the pseudorandom number generator proposed by Melià-Seguí *et al*., describing two possible attacks. Later, Garcia-Alfaro claimed that one of this attack did not hold in practice because the assumptions made by Peinado *et al.* were not correct. This letter reviews those remarks, showing that J3Gen is anyway flawed and that, without further information, the interpretation made by Peinado *et al.* seems to be correct.

## 1. Introduction

The letter by Garcia-Alfaro *et al*. [1] claims that the deterministic attack carried out by Peinado *et al*. [2] against the J3Gen PRNG (Pseudo Random Number Generator) [3,4] is not correct and seems to conclude that it is still secure. If a clear technical description is always required for a system, this becomes crucial when we talk about cryptographic proposals. More in particular, a cryptographic system should clearly specify the implementation, the range of possible parameters and the security claims for those ranges of parameters. The letter by Garcia-Alfaro *et al*. not only does not clarify some aspects that remained unclear in the original paper but it adds more doubts about the design and the results achieved with the J3Gen generator. This letter reviews such attacks and the remarks made by Garcia-Alfaro *et al*. to try to determine the security level eventually provided by J3Gen and its suitability for cryptographic purposes.

## 2. Concerning the Probabilistic Attack

Peinado *et al*. [2] presented two attacks: a probabilistic attack, based on solving linear equation systems, and a deterministic attack, based on a decimation of the output sequence. As the authors of [1] do not object anything about the former attack, one can assume that the probabilistic attacks is correct and therefore that the design of J3Gen is anyway flawed. When this probabilistic attack is applied to the set of parameters of the example described by the authors in [3], *n* = 16, *m* = 8 and *l* = 15, it only requires 240 intercepted bits to recover, on average, 7.6 of the 8 polynomials, which are supposed to be the secret information. Similarly, for other set of parameters given by the authors in [3], *n* = 32*, m* = 16 and *l* = 31, it shows that 528 output bits could be enough to recover all of the 16 feedback polynomials, and that an adversary with an output sequence of 1152 bits is able to recover all of these polynomials in half of the cases. Surprisingly, this attack is not mentioned in the letter [1] and the authors simply say that “*the closer the value of l to n, the lower the security of J3Gen to brute force attacks*”. It is true that this hinders attacks based on solving linear equations (Massey-Berlekamp analysis [5]), as the probabilistic attack does, but it would have been desirable that the letter of the authors quantified these vulnerabilities since, obviously, the results given above are not acceptable for a system intended for security purposes. On the other hand, the term “brute force attack” is usually employed for exhaustive attacks that search for a match with every possible key of the key space, and therefore its use here to refer to this kind of attacks is not very appropriate.

## 3. Concerning the Deterministic Attack

In [1], Garcia-Alfaro *et al*. object that the determinist attack is incorrect because Peinado *et al*. in [2] misinterpreted the description of the J3Gen. However, they do not provide any consistent evidence of this statement. In fact, the two main arguments that the authors use to claim that the attack is not correct are:

1. “*The list of polynomials (no matter the value of l) would never be applied as suggested in [2]*”.
2. “*The value l* = 1 *is not feasible in practice*”.

but both of them seem to be refuted if we review what Melià-Seguí *et al.* wrote about the application of the feedback polynomials and the security analysis of the parameter *l*.

- Section 3 of [3] provides, according to the authors, a detailed step by step sample execution of the scheme for the generation of 32 output bits with *n* = 16*, m* = 8 and *l* = 15. It explains (sic): “*The system starts with p*(*x*)_1_ *and outputs l* = 15 *bits* *until the TRNG module transfers a bit with value r*_0_ = 0 *to the Decoding Logic module. Then, a consecutive (but different) feedback polynomial is selected in the Polynomial Selector module, that is, p*_2_(*x*)*. This generates the next l* = 15 *LFSR shifts with p*_2_(*x*) *until the next trn is obtained. The trn value for this PRNG update is r*_1_ = 1*, hence, the Decoding Logic rotates the Polynomial Selector one position at shift 31, and another position at shift 32. Then, p*_2_(*x*) *is used 14 cycles, p*_3_(*x*) *is used one cycle, and p*_4_(*x*) *is used one cycle in this PRNG update and 14 cycles in the next PRNG update*”.

**Figure 1:**
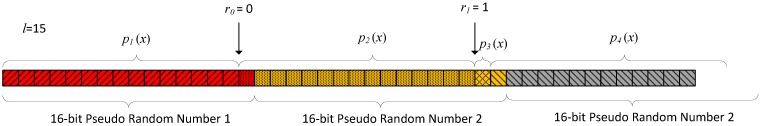
Generation of two random numbers in J3Gen.

We try to capture this with Figure 1 and Table 1 compares the case *l* = 15, described above, and *l* = 1, assumed by Peinado *et al*. Interpretation of Peinado *et al*. seems to be consistent: polynomials are applied sequentially, generating 1 or *l* bits, and as a consequence, *trn* does not have any effect when *l* = 1.

**Table 1:** Application of Primitive Feedback Polynomials in J3Gen.

*l*=15			*l*=1		
*trn*	Polynomial	Action	*trn*	Polynomial	Action
	*p*_1_(*x*)	Generate 15 (=*l*) bits		*p*_1_(*x*)	Generate 1 (=*l*) bits
*0*			*0*		
	*p*_2_(*x*)	Generate 15 (=*l*) bits		*p*_2_(*x*)	Generate 1 (=*l*) bits
*1*			*1*		
	*p*_3_(*x*)	Generate 1 bit		*p*_3_(*x*)	Generate 1 bit
	*p*_4_(*x*)	Generate 15 (=*l*) bit		*p*_4_(*x*)	Generate 1 (=*l*) bit

- Melià-Seguí *et al.* [1] claim that “*the clock signals controlling J3Gen (i.e., the LFSR clock, as well as the clock of the physical source of randomness, and the one controlling the selection of polynomials) are not synchronized*”. We can understand that activation and deactivation timings can be different but all of them should be controlled by the same single master clock. EPCGen2 tags do not have on-board clocks and they recover the clock signal from the reader’s carrier. The presence therefore of three independent clock signals requires further explanations.
- In their letter [1], the deterministic attack is said not to be feasible in practice because the value *l* = 1 is only given as a boundary and in this case J3Gen could simply output the true random sequences from the thermal-noise generator. We cannot say that the authors did not have this in mind when they described J3Gen, but at leat, it does not seem to coincide with that written in the original papers where the value *l* = 1 is not only included in the analysis, but also suggested to increase the security of the system. In particular, it says “*Depending on the level of desired security, l can be bounded by* 1 *≤ l < n*”, and follows “*Here, the attacker has to face with the uncertainty added by the feedback update rate (l). For example, using the selected parameters n* = 32 *and* *m* = 16*, if l* = 31 *it means there would be up to 4 possible solutions for each system of equations. If l* = 25 *then the possible solutions are up to 16,384, for l* = 21 *the possible solutions increase to 4,194,304, etc. The extreme case would be l* = 1 *where all 67 million primitive feedback polynomials would be equally probable*". Thus, if the authors thought that the value *l* = 1 could not be applied by practical reasons, they should have clarified the complete range of values that should be excluded by practical reasons (in the letter the authors say that it is explained in previous papers but we have not found any reference to this).

## 4. Additional Remarks

The doubts about the J3Gen generator do not stop with the implementation and the range of values, but also the provided statistical tests should be reviewed. Firstly, because the attacks described by Peinado *et al*. and even the dependence of these with the parameters, later recognized by the own authors (*l* must be not close to *n*), have not been taken into account. Secondly, because this statistical tests do not seem to be correct. For example, in [4] (Section 5.1) the authors say to conduct ten tests, running 1000 iterations per test to show (due to a misinterpretation of the EPC Gen2 standard) that the probability that any two tags simultaneously generate identical 16-bit sequence for a population up to 10,000 tags is lower than 0.04. This cannot be correct because for true random sequences of 16 bits, this probability of collision can be computed as *P* = 1 *− prob_nocollision_* where:

(1) $$prob_{nocollision}=1\times \left(1-\frac{1}{2^{16}}\right)\times \left(1-\frac{2}{2^{16}}\right)\times \ldots \times \left(1-\frac{9999}{2^{16}}\right)=1.4822\cdot 10^{-323}.$$

In fact, it can be further computed by using the following approximation (birthday paradox [6]):

(2) $$n=\sqrt{-\ln(1-P)\cdot 2^{17}}$$

that the number of sequences (*n*) required to have a probability of collision higher than 0.04 is just 74.

## 5. Conclusions

In conclusion, we regret that Garcia-Alfaro *et al*. did not use their letter to:

- clarify the aspects above mentioned,
- describe new examples of generation of random numbers that justify their arguments,
- provide clear secure ranges for the parameters of the protocol since low values of *l* are not practical,
- review all the security claims.

Without this, the use of J3Gen for cryptographic purposes remains highly inadvisable.
